# Effective heritable gene knockdown in zebrafish using synthetic microRNAs

**DOI:** 10.1038/ncomms8378

**Published:** 2015-06-08

**Authors:** Jean Giacomotto, Silke Rinkwitz, Thomas S. Becker

**Affiliations:** 1 Brain and Mind Research Institute, Sydney Medical School, University of Sydney, Camperdown, New South Wales 2050, Australia; 2 Department of Physiology, Sydney Medical School, University of Sydney, Camperdown, New South Wales 2050, Australia; 3 Present address: Institute of Molecular Bioscience, The University of Queensland, St Lucia, Queensland 4072, Australia

## Abstract

Although zebrafish is used to model human diseases through mutational and morpholino-based knockdown approaches, there are currently no robust transgenic knockdown tools. Here we investigate the knockdown efficiency of three synthetic miRNA-expressing backbones and show that these constructs can downregulate a sensor transgene with different degrees of potency. Using this approach, we reproduce spinal muscular atrophy (SMA) in zebrafish by targeting the *smn1* gene. We also generate different transgenic lines, with severity and age of onset correlated to the level of *smn1* inhibition, recapitulating for the first time the different forms of SMA in zebrafish. These lines are proof-of-concept that miRNA-based approaches can be used to generate potent heritable gene knockdown in zebrafish.

## Abstract (web summary)

Zebrafish is a model system for which for no reliable heritable gene silencing method is available. Here the authors provide a system for heritable miRNA-mediated knockdown and demonstrate tunable silencing of the *smn1* gene that recapitulate different forms of spinal muscular atrophy.

## Graphical abstract

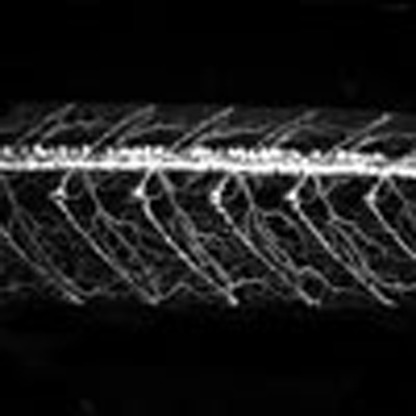

Owing to its transparency, extrauterine development, versatility for microscopy and suitability for large-scale experiments, zebrafish (*Danio rerio*) has become an attractive model for genetic and biomedical research^1^,^2^. Several reverse genetic tools, such as Zinc-finger Nucleases, TALEN and CRISPR, have been recently developed that allow to interrogate gene function and to model genetic human diseases^3^,^4^. Nonetheless, zebrafish is lacking reliable and heritable spatiotemporal knockdown tools. Knockdown through injected morpholinos can only be used for the first few days of development and is difficult to control spatiotemporally^5^. Moreover, it was recently suggested that off-target effects of morpholinos may be more prevalent than previously thought^6^. Several recent publications suggest that an alternative might come from microRNA (miRNA)-based knockdown^7^,^8^,^9^. However, this RNA interference (RNAi) approach seems to be a matter of debate in terms of efficiency and toxicity^10^,^11^,^12^.

RNAi in zebrafish has been controversial: for example, injection of long dsRNA was shown to lead to a global mRNA knockdown, suggesting induction of the interferon response^13^. Later, siRNA injections were successfully used to trigger gene knockdown^14^. However, it was demonstrated that this approach can cause nonspecific defects in zebrafish embryos^10^. This early toxicity appears to be related to nonspecific downregulation of endogenous miRNAs. In zebrafish, *miR-430* is critical for the maternal-to-zygotic transition, and high-dose siRNA injection blocks its maturation by saturating the miRNA pathway^10^,^15^,^16^. More recently, it was shown that endogenous *pri-miRNAs* could serve as a backbone to express a synthetic sequence targeting a gene of interest, as siRNA did^7^,^17^. In principle, this approach would allow to overcome toxicity problems linked to the large amount of siRNA that needs to be injected at the one-cell stage of zebrafish. In addition, the use of tissue-specific promoters in transgenes would also allow spatiotemporal control and tracking via co-expression of fluorescent markers. Although this approach would be of considerable potential, several studies suggest that it is difficult to design efficient synthetic miRNAs, and that it is also difficult to generate transgenic lines presenting potent knockdown^11^,^18^. The reasons for these limitations are not understood. So far, the zebrafish model is still lacking proof that artificial miRNA expression can be used effectively to address gene function and to generate useful transgenic models.

Spinal muscular atrophy (SMA) is an autosomal recessive human disease characterized by motoneuron loss, progressive muscle weakness and premature death^19^. SMA results from a partial loss of Survival Motor Neuron (*SMN*) gene activity. SMN is encoded by a highly conserved gene, expressed ubiquitously and lethal when fully absent. In humans, and only in humans, this protein is encoded by two nearly identical genes, *SMN1* and *SMN2* (refs 19, 20, 21). *SMN2* contains a mutation, which leads to inefficient splicing of exon 7 and to an unstable truncated protein (for review see refs 20, 21). *SMN2* is present in human in multiple copies with variable numbers between individuals. All SMA patients present full *SMN1* loss of function, partially rescued by *SMN2* expression. There are four types of SMA (I–IV) from severe to mild (OMIM 253300, 253550, 253400 and 271150), which are directly correlated to the amount of residual functional SMN protein. The zebrafish genome contains a single orthologue named *smn1*. In zebrafish, loss of function mutations of *smn1* are early lethal^22^,^23^. To mimic SMA *in vivo*, one has to downregulate the level of SMN instead of abolishing it. Efforts have been made to reintroduce human *SMN2* into the *smn1* loss of function background; however, to date this approach failed to recapitulate the hypomorphic forms of SMA in zebrafish^24^,^25^,^26^. Another approach would be to reduce SMN to a level observed in patients. This has been achieved in zebrafish through morpholino injections, revealing a critical role of SMN in motoneuron development^27^,^28^. However, this approach is limited to the first few days of the fish, is difficult to control spatiotemporally and needs to be repeated for each embryo to be studied.

Here we tested the knockdown efficiency of three miRNA-based backbones and show that they can be used to downregulate a gene of interest, but differ in potency. By targeting the *smn1* gene, we used this approach to generate new transgenic zebrafish models of SMA. These models exhibit phenotype severity that is correlated to the level of *smn1* inhibition. Thus, this strategy allows recapitulating the different forms of the disease by tuning the level of Smn protein. We achieved reduction of Smn protein by up to 90%, producing transgenic animals with early phenotypes, including motor neuron abnormalities, shorter size, abnormal swimming behaviour and premature death. Conversely, several transgenic lines with less potent *smn1* reduction developed adult onset phenotypes, including abnormal swimming behaviour, scoliosis and considerable weight loss. This is the first time that a zebrafish model offers the possibility to investigate SMA at later stages. The ease with which these heritable knockdown models can be generated opens new avenues, not only to study SMA, but also to model other diseases in which gene dosage is critical. This approach would also be useful for the study of genes that are embryonically lethal in null alleles, haploinsufficient, when spatiotemporal inhibition is desired or when several genes need to be downregulated simultaneously, such as in models of polygenic disorders.

## Results

### Generation of pME-RNAi artificial-miR-expressing constructs

**Figure 1:**
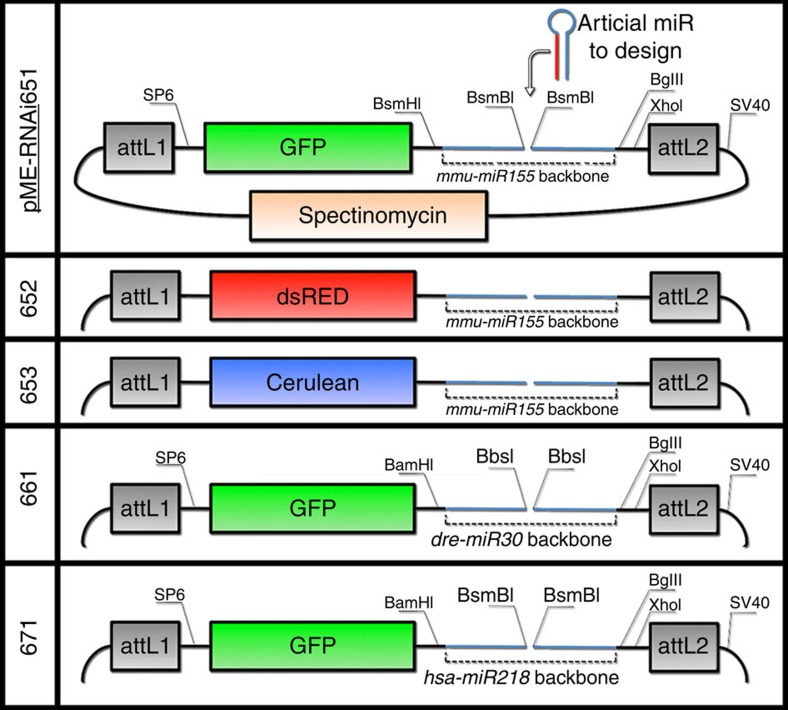
Schematics of pME-RNAi plasmids generated in this study. Three different miRNA-mediated backbones were cloned in a modified pME construct, which encodes Spectinomycin resistance and minimal sequences for RNA synthesis (SP6/SV40). pME-RNAi651 is based on a *mmu-miR155* backbone, pME-RNAi661 on a *dre-miR30* backbone and pME-RNAi671 on a *hsa-miR218* backbone. Two additional constructs, encoding dsRED or Cerulean reporters instead of GFP, were generated for the scaffold based on mmu-miR155 (named pME-RNAi652 and pME-RNAi653, respectively). Unique restriction sites BamHI, BglII and XhoI can be used to chain artificial miRNA-expressing cassettes.

**Figure 2:**
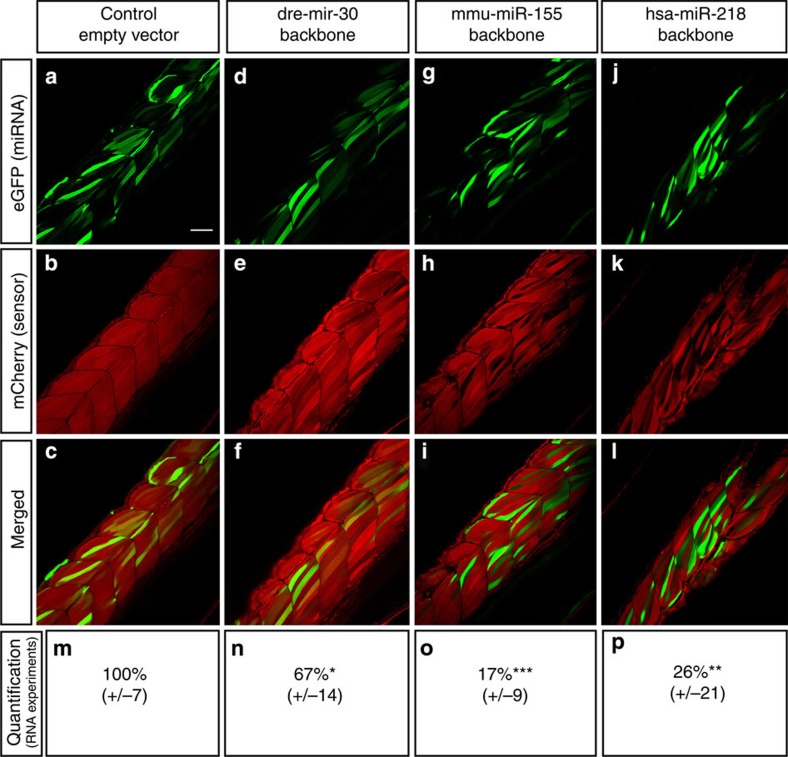
Validation of knockdown efficiency of three miRNA-expressing constructs in 52 hpf zebrafish embryos. (**a–l**) Homozygous ßactin:mCherry:sUTR embryos were injected with 30 pg of three different tol2-flanked miRNA-expressing constructs, along with transposase to promote integration. Each construct was designed to produce GFP along with the same specific artificial miRNA targeting a unique site in 3′-UTR of mCherry. Control animals were injected with the empty mmu-miR155 backbone. A minimum of 10 larvae per condition were analysed using a confocal microscope to estimate knockdown efficiency. (**m–p**) Homozygous ßactin:mCherry:sUTR embryos were injected with corresponding RNA (150 pg)—synthesized via linearized pME-RNAi clones—and raw red fluorescence was quantified, normalized and presented as percentage of control. The means of 20 embryos±s.e.m. Different from control at *P≤0.02, **P=0.0001, ***P=0.000001. Scale bar, 50 μm.

To select an appropriate vector for both *in vitro* artificial miRNA synthesis for zebrafish transient experiments and the generation of miRNA-expressing constructs for transgenesis, we generated three different plasmids compatible with the Tol2 kit components and containing minimal elements for RNA synthesis (named pME-RNAi651, pME-RNAi661 and pME-RNAi671; Fig. 1)^29^. Two constructs were generated starting from existing miR-expressing cassettes, based on the *dre-miR30* and *mmu-miR155* scaffolds^7^,^8^,^30^. The third plasmid, based on an *hsa-miR218* sequence, was made in our laboratory. To test the ability of these pME-RNAi constructs to successfully trigger genetic knockdown, we took advantage of the transgenic fish line ßactin:mCherry:sUTR, which has ubiquitous and homogeneous mCherry expression (Fig. 2). Starting from the different pME-RNAi empty backbones, we introduced compatible sequences (*pre-miRs*) that would lead to the formation of the same synthetic mature miRNA with perfect complementarity to a unique region in the 3′-untranslated repeat (UTR) of the mCherry mRNA (Fig. 1, Supplementary Figs 1 and 2).

### *miR-155* and *miR-218* backbones lead to potent knockdown

pME-RNAi651, pME-RNAi661 and pME-RNAi671 constructs with green fluorescent protein (GFP) marker followed by a synthetic *pre-miR* directed against the 3′-UTR of mCherry were cloned downstream of the ubiquitin promoter in a mini-tol2-R4R2 multisite gateway-compatible destination plasmid (Supplementary Fig. 3). Destination clones were co-injected with transposase mRNA to promote DNA integration. GFP mosaic expression was obtained as expected and allowed the tracking of artificial miR expression (Fig. 2a,d,g,j). This approach allows rapid evaluation of mCherry knockdown efficiency within a single fish larva by comparing cells expressing synthetic miRs (GFP-positive) to cells expressing little or no miRNA (GFP-negative; Fig. 2a–l). All three *pri-miR* backbones led to a visible cell-specific downregulation of mCherry accumulation. However, the backbone based on *dre-miR30* achieved only weak red fluorescence inhibition compared with *mmu-miR155* or *hsa-miR218* backbones. In addition to these experiments, RNAs containing anti-mCherry synthetic *pri-miR* were synthesized from the corresponding linearized pME-RNAi constructs using the SP6 promoter (Fig. 1). Each RNA batch was injected at 150 pg per embryo and raw fluorescence was quantified at 30-h post fertilization (hpf). All three backbones led to a significant reduction in red fluorescence (Fig. 2m–p). According to the cell-specific observations above, *miR218* and *miR155* backbones led to potent global knockdown with ∼74 and 83% reduction in red fluorescence, respectively, while the *miR30* backbone reduced fluorescence modestly by ∼33%.

### Targeting the 3′-UTR leads to potent knockdown

**Figure 3:**
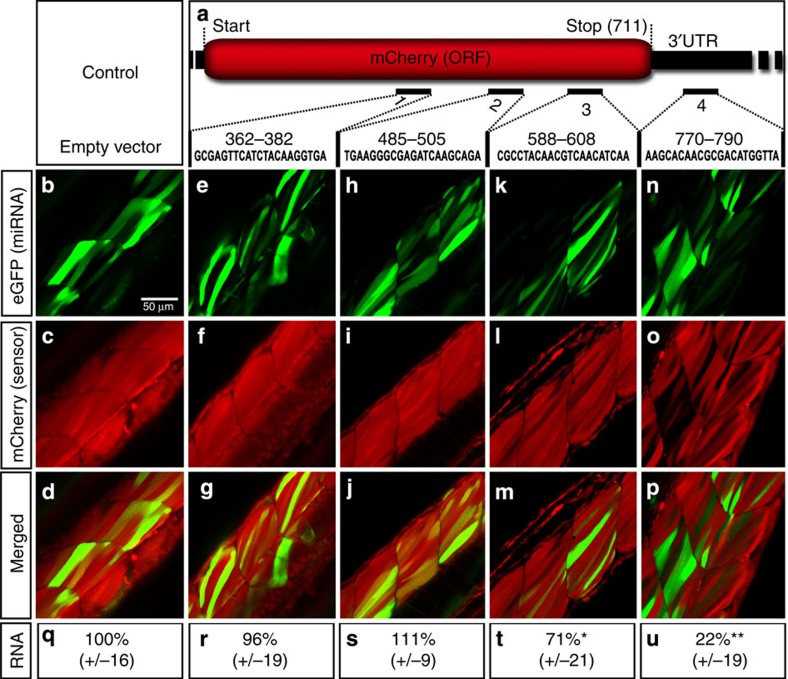
Evaluation of the effect of target sequence/position on knockdown efficiency in 52 hpf zebrafish embryos. (**a**) Schematic representation of mCherry mRNA produced in the ßactin:mCherry:sUTR zebrafish line, as well as in artificial miRNA targets. (**b**–**p**) Embryos with ubiquitous mCherry expression were injected with transposase and 30 pg of tol2-flanked miRNA-expressing constructs (mmu-miR155 backbone), which were designed to produce artificial miRNA targeting different sites of the mCherry mRNA. A minimum of 10 larvae per condition were analysed in order to estimate knockdown efficiency. (**q**–**u**) Homozygous ßactin:mCherry:sUTR embryos were injected with corresponding RNA (150 pg), synthesized via linearized pME-RNAi clones, and raw red fluorescence was quantified, normalized and presented as percentage. The means of 10 embryos±s.d. Different from control at *P=0.05, **P=0.000001. Scale bar, 50 μm.

We next tested the potential of the *miR155* backbone to trigger knockdown when the open reading frame (ORF) was targeted (Fig. 3). In addition to the 3′-UTR target site, we defined three sites in the ORF of mCherry mRNA (Fig. 3a, Supplementary Figs 1 and 2). All four sequences, driven by the ubiquitin promoter, were injected along with transposase mRNA into zebrafish embryos expressing the reporter mCherry protein (Fig. 3a–p). Again, targeting the 3′-UTR led to a visible cell-specific mCherry knockdown (Fig. 3n–p), while two target sites in the ORF did not result in any obvious visible effect (Fig. 3e–j), and a third site inhibited mCherry expression only slightly (Fig. 3k–m). To confirm these observations, RNAs encoding each artificial miRNA were synthesized from pME-RNAi clones and injected at 150 pg per embryo. Artificial miRNA targeting the 3′-UTR region reduced red fluorescence by 78%, while only one miR directed against the ORF (target site 3) was able to significantly downregulate mCherry expression by ∼29% (Fig. 3q–u).

### Stable and heritable miRNA-mediated knockdown of *dre*-*smn1*

**Figure 4:**
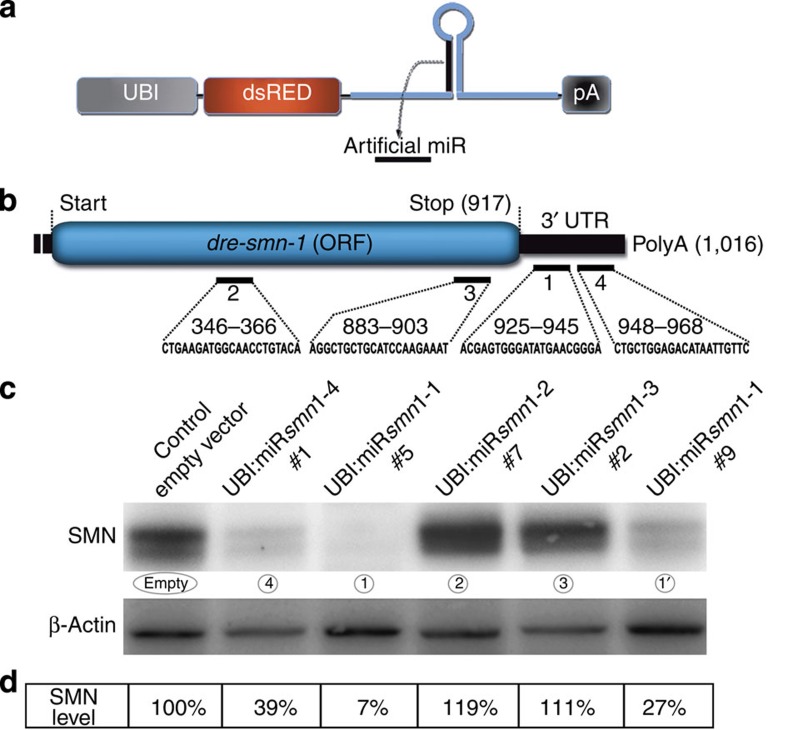
Generation and analysis of zebrafish transgenic lines expressing artificial microRNAs against different ***smn1*****target sites**. (**a**) schematic representation of miR-RNAi construct used to release artificial miRNA against *smn1*, based on the *mmu-miR155* scaffold. (**b**) Schematic view of *dre-smn1* transcript and corresponding target sites. Artificial anti-*smn1* miRNA (miRsmn1) target sequences are annotated from 1 to 4. (**c**) Western blot analysis of whole 72hpf-transgenic larvae lysate revealing endogenous SMN protein. (**d**) Western blot band quantification. SMN band intensity is expressed as the percentage of control. UBI:miRsmn1-X, with X between 1 and 4, corresponds to *smn1* target position number.

To test the potential of this approach to knockdown endogenous gene expression and to try to mimic SMA in zebrafish, synthetic miRNAs were designed to target the *dre-smn1* gene (Fig. 4). Four targets were selected, with two in the *smn1* 3′-UTR and two in the ORF, named miRsmn1-1 to -4 (Fig. 4b and Supplementary Fig. 2). All miRsmn1-expressing constructs were based on the *mmu-miR155* scaffold fused to dsRED (pME-RNAi652), and were cloned downstream of the ubiquitin promoter in a mini-tol2-R4R2 destination plasmid (Fig. 4a). To generate transgenic lines, the corresponding DNA constructs (that is, UBI:miRsmn1-1 to -4) were microinjected into one-cell stage hsa-miR218-2:GFP zebrafish embryos, a transgenic line that specifically expresses GFP in motor neurons^31^. Several founders were identified for each construct, and then sorted based on strong dsRED expression in F1 embryos. The highest expressing founders for UBI:miRsmn1-2 to -4 constructs were then outcrossed to establish F1 transgenic lines and were named as follows: UBI:miRsmn1-2#7, UBI:miRsmn1-3#2 and UBI:miRsmn1-4#1 (Fig. 4c). Two founders and two independent F1 transgenic lines were generated for the miRsmn1-1 construct, named UBI:miRsmn1-1#5 and #9 (Fig. 4c). F1 transgenic lines were further evaluated in regards to the number of transgene insertions. Each F1 transgenic line used gave birth to a ratio of close to 50% positive dsRED embryos when outcrossed with wild-type animals, suggesting the presence of a single insertion (or at least insertion(s) in a single chromosome). In order to analyse *smn1* knockdown efficiency, F1 animals were incrossed and the brightest 20 embryos per cross were sorted for analysis. The larvae were killed at 72 hpf to extract whole-lysate proteins. Smn protein was revealed and quantified in each genetic background via western blot and image analysis (Fig. 4c,d). No significant difference was observed for artificial miRs targeting the *smn1* ORF, while a strong reduction was detected for miRs targeting the 3′-UTR. Using image j band intensity measurement, Smn was reduced by 61% in UBI:miRsmn1-4#1, by 73% in UBI:miRsmn1-1#9 and by 93% in the UBI:miRsmn1-1#5 zebrafish line. Similar results were obtained at 6-days post fertilization (dpf), suggesting that *smn1* knockdown was stable over time (Supplementary Fig. 4). These results show that artificial miRNA-expressing constructs can be used to generate transgenic zebrafish that undergo potent gene knockdown as we achieved the reduction of Smn protein by up to 90%.

### *smn1* knockdown transgenic fish show hallmarks of SMA

**Figure 5:**
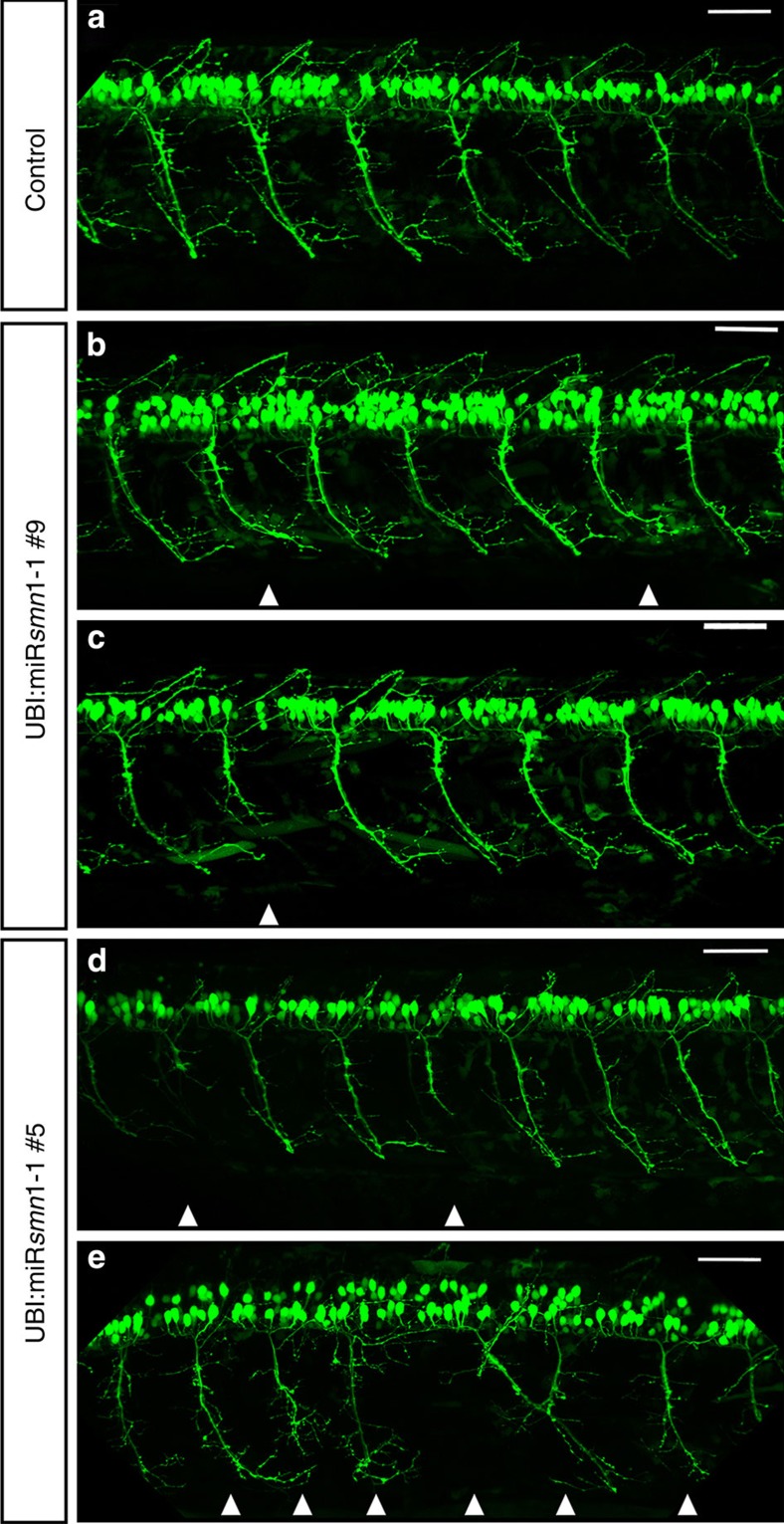
Representative images of 52 hpf zebrafish larvae expressing GFP in motor neurons±***smn1*** **knockdown**. Lateral views of (**a**) control (empty vector) and (**b–e**) transgenic zebrafish larvae expressing artificial miR (targeting *smn1* 3′-UTR). Compared with control animals, transgenic larvae expressing miRsmn1 present abnormal motor neuron development, including abnormal branching, short axons as well as pathfinding errors (white arrowheads). Transgenic animals expressing artificial miRNA against the ORF (targets 2 and 3) do not show obvious motor neuron abnormality. Scale bar, 50 μm.

**Figure 6:**
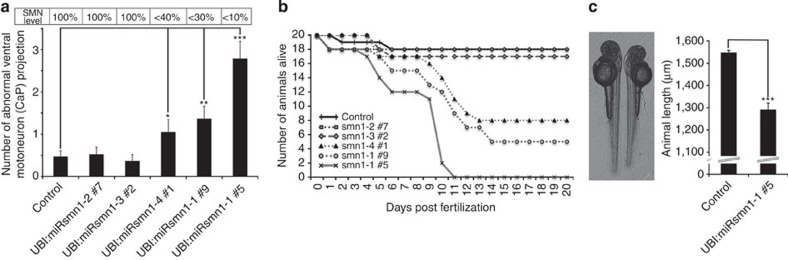
Phenotype severity is correlated with the level of SMN1 inhibition. (**a**) Number of ventral motor neuron (CaP) abnormalities observed per side of 52 hpf larvae (short axons, abnormal branching, pathfinding error and/or absence of cell body). Raw red fluorescence evaluation of each population (reflecting transgene expression) is shown in Supplementary Fig. 4a. (**b**) Survival assay of different transgenic lines expressing artificial miRNA against *dre-smn1*. (**c**) Animal size comparison between 52 hpf control (empty vector) and 52 hpf UBI:miRsmn1-1#5 transgenic lines (compared with control, other miRsmn1-expressing lines do not present significant difference). The means of 20 larvae±s.e.m. Different from control at *P≤0.04, **P=0.01, ***P=0.0001.

We next tested whether our transgenic lines presented signs of SMA. It has been shown previously via morpholino injection that transient *smn1* knockdown leads to motor neuron malformations^27^. We started by examining primary motor neuron development to see whether this phenotype was recapitulated in miRsmn1-expressing fish. As above, F1 animals were incrossed and the 20 brightest embryos per cross were sorted out for analysis. Motor neuron axonal projections were examined at 30 hpf, 52 hpf and 5 dpf. There are four types of primary motor neurons, including Caudal Primary (CaP) motor neurons that project long axons ventrally (for review see ref. 32). We only quantified CaP motor neuron projection abnormalities. As expected, animals that express miRsmn1-2 and -3, which target the *smn1* ORF, did not show any significant motor neuron projection aberration at 52 hpf when compared with controls. However, fish expressing artificial miRNA directed against the *smn1* 3′-UTR had CaP motor neuron abnormalities at 52 hpf, including short axons, abnormal branching, as well as pathfinding errors (Fig. 5 and Fig. 6a). F2 transgenic UBI:miRsmn1-1#5 embryos, which present ∼90% Smn1 reduction, were the most affected at 52 hpf with 2.8 (±0.4) abnormal CaP motor neuron projections per side analysed. UBI:miRsmn1-1#9 and UBI:miRsmn1-4#1, with ∼70% and 60% Smn1 reduction, respectively, presented with 1.4 (±0.3) and 1.1 (±0.3) aberrant CaP motor neuron projections per side. In addition, anti-SV2 and anti-α-tubulin immunostaining performed at 52 hpF on F2 transgenic animals confirmed the *in vivo* results presented above (Supplementary Figs 5 and 6).

In addition to motor neuron phenotypes, *smn1* miR-mediated knockdown fish died prematurely (Fig. 6b). As for motor neuron phenotypes, F2 UBI:miRsmn1-1#5 larvae were the most affected, with no larvae that survived beyond 11 days. No other obvious anatomical abnormality was detected in any miRNA-expressing transgenic lines, except for UBI:miRsmn1-1#5 F2 larvae, which were significantly shorter starting from 52 hpf (Fig. 6c). The absence of other malformations suggests that the level of artificial miRNA expression in the line tested does not induce toxicity such as observed after siRNA injection^10^.

As SMA impairs motor function in human, swimming behaviour was investigated. Animals were loaded into 24-well plates and spontaneous swimming behaviour was recorded for 10 min. No significant difference was detected with the different transgenic lines, except for F2 UBI:miRsmn1-1#5 animals, which showed strong motor impairment starting at 5 dpf (Supplementary Fig. 7 and Supplementary Movie 1). Compared with control, overall distance swum by these larvae, as well as overall speed, was dramatically reduced (Supplementary Fig. 7b,c). Thus, in addition to motor neuron abnormalities, F2 UBI:miRsmn1-1#5 animals had poor motor function.

### *hsa-SMN1* rescues *smn1* knockdown phenotypes

**Figure 7:**
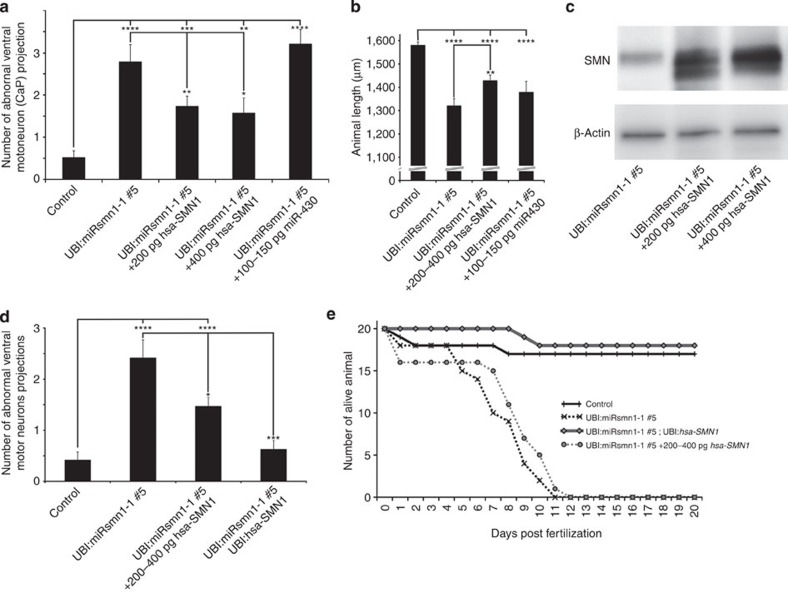
Phenotypic rescue of UBI:miRsmn1-1#5 transgenic line. (**a**) Number of ventral motoneuron (CaP) abnormalities observed per side of 52 hpf larvae. miRsmn1-5-expressing animals were injected with either *hsa-SMN1* RNA or miR-430 to test their capacity to rescue the observed motoneuron phenotype. (**b**) Animal size comparison between 52 hpf control (empty vector) and 52 hpf UBI:miRsmn1-1#5 transgenic lines±*hsa-SMN1* RNA or miR-430 injection. (**c**) Western blot analysis of whole 56 hpf UBI:miRsmn1-1#5 larvae lysate (±*hsa-SMN1* RNA) revealing *dre*-SMN and *hsa*-SMN protein. (**d**) Number of ventral motor neuron (CaP) abnormalities observed per side of 52 hpf larvae. Rescue experiment comparing RNA injection versus ubiquitous transgenic expression of *hsa-SMN1* (see Supplementary Fig. 5 for strategy used). (**e**) Survival assay comparing control (empty vector) versus UBI:miRsmn1-1#5±*hsa-SMN1* RNA injection or ubiquitous transgenic expression of *hsa-SMN1*. Means of 20 larvae±standard error of the mean. Different from control at *P≤0.04, **P=0.02, ***P=0.001, ****P=0.0001.

In order to validate these new zebrafish SMA models, we investigated the potential of human *SMN1* to rescue the phenotypes presented above. First, we injected *hsa-SMN1* mRNA into F2 UBI:miRsmn1-1#5 embryos at 200 and 400 pg. As before, the brightest 20 embryos per condition were selected for further analysis. Both concentrations partially rescued the CaP motor neuron phenotype observed at 52 hpf in F2 UBI:miRsmn1-1#5 animals. The number of abnormal CaP motor neuron projections in F2 UBI:miRsmn1-1#5 was reduced from 2.8(±0.4) to 1.7(±0.2) and 1.6(±0.4) after 200 and 400 pg *hsa-SMN1* mRNA injection, respectively (Fig. 7a). Similarly, *hsa-SMN1* mRNA partially rescued the reduced length of F2 UBI:miRsmn1-1#5 animals (Fig. 7b). Larvae were killed at 60 hpf to extract whole-lysate protein. Western blot analysis against SMN1 confirmed that *hsa-SMN1* mRNA was translated into detectable SMN1 protein (Fig. 7c). These results demonstrate that the observed phenotypes were specific to *smn1* knockdown. However, because it has been shown that an excess of artificial small RNA can flood the endogenous miRNA pathway and lead to toxicity by repressing miR-430 maturation, a possible role of *miR-430* was investigated^10^,^15^,^16^. A synthetic form of *miR-430* was injected at ∼100–150 pg. No significant effect was detected on motor neuron development and overall length of 52 hpf F2 UBI:miRsmn1-1#5 animals (Fig. 7a,b).

**Figure 8:**
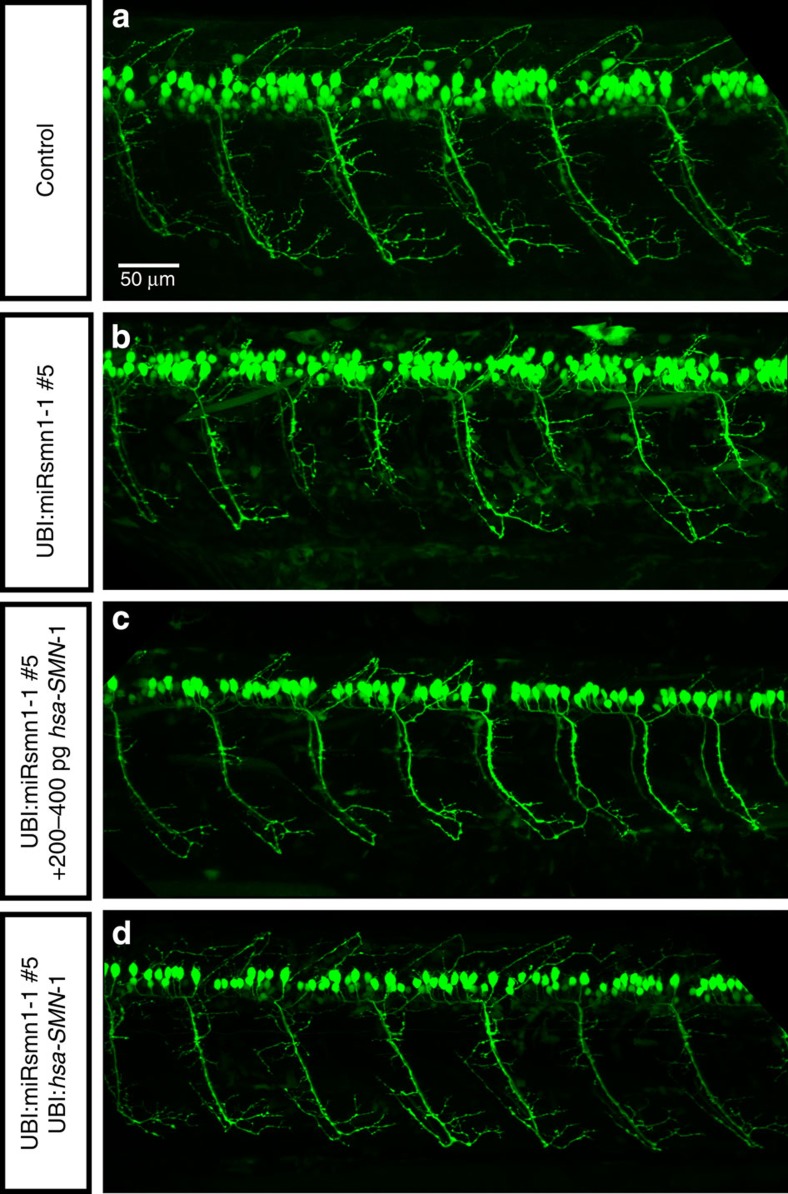
*hsa-SMN1* rescues the *dre-smn1* knockdown motor neuron phenotype. Representative images of 52 hpf zebrafish larvae expressing GFP in motor neurons. Lateral views of (**a**) control (empty vector) transgenic animals. (**b**–**d**) transgenic UBI:miRsmn1-1#5 zebrafish line (presenting ∼90% downregulation of SMN), with *hsa-SMN1* RNA injection (**c**) or *hsa-SMN1* ubiquitous expression via integrated transgene (**d**; see Supplementary Fig. 7 for strategy used). Scale bar, 50 μm.

In addition to these transient experiments, a transgene carrying *hsa-SMN1* under the control of the ubiquitin promoter was introduced into the F1 UBI:miRsmn1-1#5 transgenic line as described in Supplementary Fig. 8. A founder F0 UBI:miRsmn1-1#5; UBI:hsa-*SMN1*(clmc2:GFP) was outcrossed with F1 UBI:miRsmn1-1#5. Embryos were separated into two batches, one injected with 200–400 pg *hsa-SMN1* RNA to serve as positive control and one kept without treatment (Supplementary Fig. 8). Embryos were then sorted as described schematically in Supplementary Fig. 8 to obtain untreated UBI:miRsmn1-1#5 embryos (negative control), UBI:miRsmn1-1#5 embryos treated with 200–400 pg *hsa-SMN1* RNA (positive control) and UBI:miRsmn1-1#5; UBI:*hsa-SMN1*(cmlc2:GFP) animals. Confirming the transient experiments, both the presence of *hsa-SMN1* RNA or the integrated UBI:*hsa-SMN1*(cmlc2:GFP) transgene led to a reduction in the CaP motor neuron phenotype at 52 hpf. However, while injected mRNA only partially rescued *smn1* knockdown, transgenic *hsa-SMN1* expression resulted in complete rescue (Figs 7d and 8). Larvae expressing *hsa-SMN1* were also indistinguishable from wild type in terms of overall anatomy and length at 52 hpf. Finally, the presence of the transgene restored a normal survival rate, while injection of mRNA did not extend the life of the affected larvae (Fig. 7e). These results confirm that the defects observed in UBI:miRsmn1-1#5 were specific to *smn1* knockdown, and that Smn protein is critical to early motor neuron development in zebrafish and also for survival, presumably independently of its early role in motor neuron formation.

### *smn1* knockdown recapitulates the hypomorphic forms of SMA

We generated several independent transgenic lines presenting phenotypes with different degrees of severity, proportional to the level of *smn1* reduction (Figs 4, 5, 6). There are four types of SMA with outcomes linked to the amount of residual functional SMN. Depending on the type of the disease, patients present with early to late/adult motor neuron disease along with very short- to normal lifespan. Thus, our miR-mediated knockdown approach allows to better recapitulate these conditions. For example, F2 UBI:miRsmn1-1#5 animals are comparable to the severe form of the disease with early phenotypes, and death early in life. Interestingly, F1 UBI:miRsmn1-1#5, which did not show increased death at the larval stage or strong early motor neuron phenotype, developed adult phenotypes starting from 3 months post fertilization, including marked weight loss, scoliosis and abnormal swimming behaviour (Supplementary Fig. 9 and Supplementary Movie 2). It is noteworthy that double transgenic UBI:miRsmn1-1#5; UBI:*hsa-SMN1*(cmlc2:GFP) animals—used in the rescue experiment above—did not show any obvious defect at adult stages, confirming the specificity of these adult phenotypes. Similar to intermediate forms of SMA, F2 UBI:miRsmn1-1#9 and F2 UBI:miRsmn1-4#1 showed mild early phenotypes with better survival outcome than F2 UBI:miRsmn1-1#5 (Fig. 6). However, no sorted fish survived more than 3 months and reached the adult stage. Thus, using miR-mediated knockdown, it is possible to mimic the different hypomorphic states of SMA in zebrafish.

## Discussion

We showed here that the use of synthetic miRNA is a viable approach to knockdown a gene of interest in zebrafish. We first generated pME-RNAi clones that present the advantage of allowing RNA synthesis for rapid knockdown validation and that are also compatible with the Tol2 kit (Fig. 1)^23^,^29^. We tested three *pri-miR* backbones and showed that they are able to trigger RNAi. Surprisingly, in our hands, backbones based on *pri-dre-miR30*, which was previously used in zebrafish studies, had a very limited effect (Fig. 2)^7^,^8^. However, only one target was used to test and to compare the different constructs. More experiments may have to be performed to definitively conclude on the efficiency of each backbone to trigger knockdown. We suspected that each *pri-miR* sequence would behave differently depending on the artificial miRNA sequence that has to be processed. The sequence used in our experiments may not be compatible with the *pri-dre-miR30* sequence. Unfortunately, it is currently difficult to predict which backbone/*pri-miR* would be the most appropriate to process a particular artificial miR sequence. Nonetheless, *pri-dre-miR30* still presents limitations in terms of use. The restriction site used to insert artificial *pre*-miRs—that is, 2 × BbsI—does not allow directional cloning and, more problematically, is present in several fluorescent markers, hampering the use of mCherry or dsRED markers for instance.

In addition to backbone-dependent limitations, our data also suggest that the choice of the target sequence is critical. For example, it is difficult to design an efficient synthetic miR that targets the ORF, and those that triggered an RNAi response only showed poor knockdown. Interestingly, the literature tends to confirm this phenomenon, not only in zebrafish but also in mouse and rat, but not in *C. elegans*^7^,^8^,^9^,^33^,^34^,^35^. While targeting the ORF in these organisms is difficult for reasons not understood to date, we found that using 3′-UTRs as target led to efficient gene silencing with both our sensor construct and *smn1*, supporting the idea that one should prioritize the selection of targets inside the 3′-UTR of the selected gene whenever possible. If there is no other choice than targeting the ORF, several miRs may have to be selected and carefully validated to select a potent one, if any can be found. Recently, Dow *et al*.^33^ empirically defined some rules to help selecting such artificial miRNAs; these rules apply for mouse, but may work in zebrafish as well. As the pME-RNAi clones have been designed to easily chain artificial miRNAs, we strongly recommend also multiplying the copy numbers to increase potency.

Finally, as an improvement, the addition of an intronic sequence into the pME-RNAi constructs may in principle increase knockdown efficiency as well as the stability of the fluorescent marker produced along with the artificial miRNA(s)^34^. This might be critical if one wants to chain more than four miRNAs into the same backbone, as our recent work shows that chaining miRNAs does increase knockdown efficiency but dramatically decreases fluorescent expression (unpublished results).

Using transgenic artificial miRNA expression, we generated several lines that present heritable and potent (up to 90%) *smn1* gene knockdown. To our knowledge, this is the first evidence that miR-mediated knockdown in zebrafish can lead to the generation of useful transgenic animals for biomedical research. Indeed, several attempts demonstrated that heritable gene inhibition using artificial miR could be achieved, but with a level of inhibition that is not potent enough to suit traditional genetic studies^36^,^37^. A reason that might explain these results is that researchers often focused on the ORF to select target sequences. As explained above and supported by our results, the ORF is difficult to target in zebrafish or mouse^33^. Our success may result from the use of the *smn1* 3′-UTR, or, alternatively, *smn1* might be exceptionally sensitive to this knockdown approach.

Using the technique described here, we generated new transgenic models of SMA. There are four different types of SMA in human, with onset and severity correlated to the residual SMN protein level^20^,^21^. To date there is no zebrafish model that recapitulates these hypomorphic states, and while an *smn1* mutation has been recently identified in zebrafish, total absence of SMN does not reflect SMA conditions^22^,^23^. Efforts are now under way to introduce human *SMN2*, as was performed in the mouse^23^,^24^,^25^. However, even if the corresponding models might be useful to study factors that would act on *SMN2* stability and/or synthesis, they failed to mimic the different hypomorphic forms of SMA in zebrafish^24^,^25^. Using synthetic miRNA expression against *smn1*, one can now easily generate allelic series of transgenic lines that better mimic SMA in zebrafish. We have already generated animals with very early phenotypes to animals with adult onset, offering new possibilities to investigate SMA biology. It is also now straightforward to interrogate this disorder tissue specifically by restricting knockdown to muscle or to specific neuronal cell populations.

There is no cure for SMA, and patients are still awaiting palliative treatment that would improve their day-to-day life, especially with respect to motor function. Beneficial drugs might already exist in the current pharmacopeia^35^,^38^,^39^. However, finding such drugs is a challenge as there is no SMA model suitable for large-scale drug screening; with the exception of cell lines that can be used to find drugs increasing human *SMN2* gene activity^40^. Indeed, mouse models are not compatible with large-scale drug screening experiments. Zebrafish is, but not the current, SMA model. For example, homozygous null *smn1*^(−/−)^ animals are not viable, while heterozygous fish are phenotypically normal^22^,^23^. Consequently, to generate homozygous larvae to screen, one has to incross heterozygous fish, which will result in 75% larvae with no phenotype and that are impossible to sort out before analysis. This drawback makes large-scale screening experiments difficult and also hampers the workflow of traditional research experiments. Finally, incorporation of human *SMN2* failed to rescue *smn1*^(−/−)^ lethality^24^,^25^.

MiR-mediated knockdown zebrafish models of SMA would be a valid option for drug screening. Indeed, this approach allows to generate animals that are relevant to SMA and that present the advantage of expressing a fluorescent marker, allowing sorting out the affected embryos before experiments. Moreover, the most affected embryos in our study (F2 UBI:miRsmn1-1#5) presented with strong motor phenotypes (Supplementary Fig. 7, Supplementary Movie 1). Such a motor phenotype is relatively easy to use in large-scale experiments, and presumably relevant for seeking beneficial drugs that would improve patient lives^2^,^41^,^42^.

In conclusion, we demonstrate here that promoter-driven synthetic miRNA expression can be used to trigger potent heritable gene silencing in zebrafish. It is probable that miRNA-mediated knockdown will never be able to reproduce a complete null condition. However, miRNA-mediated knockdown may in some scenarios, such as for SMA disease, be a more flexible option for interrogating gene function. Indeed, the ease to restrict their effect tissue specifically as well as the possibility to target specific isoforms or to generate hypomorphic states would enrich the study of gene function and human diseases in which gene dosage is critical. For example, many gene knockouts are embryonically lethal but fully viable as heterozygotes. It has also been reported that complete loss of function often leads to molecular compensation, hiding the role of the deleted gene^43^. Partial knockdown of protein function may also mimic chemical treatments more closely than complete abolition of protein function. Finally, it is relatively easy and inexpensive to generate such models. In the future, this approach may also be used to study diseases with polygenic inheritance—that is, cases where more than one gene has to be reduced in expression to generate a phenotype^44^.

## Methods

### Zebrafish maintenance and transgenic lines

Adult Zebrafish and embryos were maintained by standard protocols approved by the University of Sydney Animal Ethics Committee.

### Generation of pDONR224 plasmid

Tol2kit-compatible pME (Middle Entry) clones are usually generated via pDONR221 plasmid and/or present kanamycine resistance gene. The kanamycine resistance gene contains a BsmBI site, hampering the use of the miRNA-expressing cassettes presented in Fig. 1. To generate a compatible pDONR clone, we started from pDONR223 plasmid (gift of Dr David Hill), which presents spectinomycin resistance. To eliminate an XhoI site that would interfere with miRNA-chaining procedures, pDONR223 was digested with XhoI and dephosphorylated (Antartic phosphatase). Oligos 78-Forw-xhoIdelete and 79-Rev-xhoIdelete were annealed and cloned into linear pDONR223 to generate pDONR223-xhoI (primers are listed in Supplementary Fig. 1). To introduce a polyA signal sequence downstream of the attL2 recombination site, pDONR223-xhoI was digested by EcoRV and dephosphorylated. A SV40-polyA signal sequence was amplified from pCS2p+ using oligonucleotides 72-SV40-Forw and 73-SV40-Rev. The PCR product was purified, digested with EcoRV and inserted into linearized pDONR223-xhoI to generate pDONR 224.

### pME-RNAi651, 652 and 653 plasmid generation

*mmu-miR155*-based plasmid: ‘pcDNA 6.2 GW/EmGFP-miR', containing artificial miRNA-expressing *mmu-miR155* backbone, was provided by Dr Donald Love^9^. The eGFP:*mmu-miR155* cassette was transferred into pDONR224 using BP clonase reaction (following the manufacturer's instructions). The resulting plasmid was named pME-RNAi641. eGFP in pME-RNAi641 was exchanged with dsRED or Cerulean sequences to generate pME-RNAi642 and pME-RNAi643, respectively. For this purpose, pME-RNAi641 was digested by DraI and dephosphorylated. The dsRED sequence was amplified from ‘myoD:*itr1*:dsRED' (unpublished construct) using primers 41-dsREDForward and 42-dsREDreverse, and the PCR product was exchanged with the eGFP cassette of pME-RNAi641 to generate pME-RNAi642. Cerulean sequence was amplified from pCH-mcs (gift from Dr L. Nonet) using primers 66-ceruleanforward and 67-ceruleanreverse; PCR product was digested with DraI and exchanged with an eGFP cassette to generate pME-RNAi643. In order to introduce SP6 minimal promoter sequence for RNA synthesis, pME-RNAi641 to 643 were digested by AflII/NcoI and gel-purified. A PCR product containing the SP6 minimal promoter was synthesized using primers 80-M13extended and 81-pME-SP6intoduction using pME-RNAi641 as matrix. PCR product was digested by AflII/NcoI and cloned into purified linear pME-RNAi64X constructs; resulting plasmids were named pME-RNAi651, pME-RNAi652 and pME-RNAi653 (Fig. 1). pME-RNAi651 to 653 as well as pME-RNAi641 to 643 are available on request.

### pME-RNAi661 plasmid generation

*mmu-miR30*-based plasmids: Artificial miRNA expressing the *dre-miR30* cassette was amplified from pCS2-mir-linker (gift from Dr Min Deng)^7^. Primers used (52_Forw_miR30 and 53_Rev_miR30) were designed to add BamHI in 5′ of the *dre-miR30* cassette, as well as BglII and XhoI in 3′, thus allowing easy chaining of *dre-miR30* (Fig. 1). Both the PCR product and pME-RNAi651 plasmid were digested by BamHI and XhoI to exchange the *mmu-miR155* backbone with the *dre-miR30* one (plasmid was purified before ligase reaction). The resulting plasmid was named pME-RNAi661 (Fig. 1).

### pME-RNAi671 plasmid generation

*hsa-miR218*-based plasmids: An artificial miRNA-expressing *hsa-miR218* cassette was generated in our laboratory. pME-RNAi651 was digested by BamHI/XhoI and gel-purified to eliminate *mmu-miR155*. *Pri-hsa-miR218-2* was amplified and cloned into pME-RNAi651 in two steps to allow the insertion of a 2 × BsmBI repeat at the *pre-miR218* position (Fig. 1). In a first step, primers 36_miR218Forw-5-BamHI and 37_miR218Rev-middle-SpeI-XhoI were used on human DNA to generate the 5′-part. The PCR product was digested with BamHI/XhoI and cloned into purified pME-RNAi651. In a second step, the resulting plasmid was digested with SpeI/XhoI and gel-purified. Primers 38_miR218Forw-middle-SpeI and 39_miR218Rev-3-BglII-XhoI were used to amplify the 3′-part of pri-miR218 including another BsmBI restriction site. The PCR product was digested with SpeI/XhoI and cloned into the previously digested plasmid. The final construct was named pME-RNAi671 (Fig. 1).

### miRNA target site selection

To select target sequences, we mainly used the BLOCK-IT RNAi Designer website from Invitrogen that is optimized for artificial miRNA-mediated knockdown (http://rnaidesigner.lifetechnologies.com/rnaiexpress/setOption.do?designOption=mirna&pid=8945441149181672314). Among other tools and websites, we used also GenScript's siRNA Design Center (www.genscript.com/design_center.html), siSearch (sidirect2.rnai.jp/), IDT SciTools RNAi Design (http://sg.idtdna.com/Scitools/Applications/RNAi/RNAi.aspx/), Target Finder supplied by Ambion (http://www.ambion.com/techlib/misc/siRNA_finder.html), Dharmacom RNAi design centre (http://dharmacon.gelifesciences.com/design-center/) and siSPOTR tools (https://sispotr.icts.uiowa.edu/).

Targetscan Fish (http://www.targetscan.org/fish_62/) is also useful to obtain complete and accurate 3′-UTR sequences of a gene of interest in zebrafish.

### Oligo-miRNAs (artificial *pri-miRNA*) design

Each pME-RNAi plasmid is designed to easily insert an artificial *pri-miRNA* on the basis of BsmBI or BbsI restriction sites (Fig. 1 and Supplementary Fig. 10). A schematic representation describing the structure of each *pri-miRNA* and the corresponding oligonucleotides are listed in Supplementary Fig. 10. No specific modifications are required to synthesize these oligonucleotides.

### Oligo-miRNAs (artificial *pri-miRNA*) annealing

In all, 5 μl of 200 μM of each Forward and reverse oligonucleotides were mixed in a 20 μl reaction including 2 μl NEB 10 × buffer2. The mixture was heated to 95 °C for 5 min using a thermocycler and left to cool down in the thermocycler's plate for 30 min. Samples were mixed and briefly spun down before being diluted 5,000-fold in water at room temperature. Diluted samples were stocked at room temperature until ligation.

### Oligo-miRNAs (artificial *pri-miRNA*) cloning

The different pME-RNAi plasmids were digested with BsmBI or BbsI and gel-extracted/purified. Linearized pME-RNAi backbones were stored at −20 °C at 300 ng μl^−1^. To insert a specific artificial *pri-miRNA*, 10 ng of linearized pME-RNAi construct was mixed with 4 μl of the 1:5,000-diluted ds-oligos (artificial *pri-miR*) and ligated following the manufacturer's instruction (NEB T4 DNA ligase).

### DNA injection and selection of transgenic lines

To integrate DNA constructs into the zebrafish genome, 1 nl of a mix containing 30 ng μl^−1^ of DNA of interest plus 25 ng μl^−1^ of Transposase mRNA and phenol red were injected at the one-cell stage.

### Western blot analysis and quantification

For each condition, 20 embryos were dechorionated using pronase (1 mg ml^−1^, 10 min at 28 °C) and rinsed three times in calcium-free Ringer's solution. Yolks were separated from embryos by pipetting up and down using Pasteur pipettes. Samples were centrifuged 5 min at 200*g*. Pellets were washed in cold PBS and centrifuged for 5 min at 200 g, 4 °C. Pellets were resuspended in 20 μl of cold RIPA buffer (50 mM Tris-HCL pH 7.4, 150 mM NaCl, 1 mM EDTA, 1% Triton X-100, 1% sodium deoxycholate, 0.1% SDS) containing protease inhibitors (Roche). Samples were centrifuged 20 mn at 2,000*g*, 4 °C. Supernatants were collected and stored at −80 °C for western blot analysis. The following antibodies were used: Anti-SMN1 (MANSMN12, 2E6, 1:5,000; Glenn E. Morris, Developmental Studies Hybridoma Bank) and Anti-β-Actin antibody (Sigma-Aldrich, Catalogue number A5441-.2ML, 1:5,000). Western blot band intensity measurements were performed in ImageJ and normalized using β-Actin band intensity. Uncropped blots are shown in Supplementary Fig. 11.

### Immunohistochemistry

Dechorionated zebrafish embryos/larvae were fixed in 4% paraformaldehyde for 3 h at room temperature or overnight at 4 °C. Animals were washed three times in PBT (1 × PBS+0.8% Triton X) and then gradually transferred into 100% methanol, via a 5-min step at 50/50% PBT/methanol. Animals were stored in −20 °C if required. Animals were then rehydrated for 5 min in 50/50% PBT/methanol and three times in PBT for 5 min. Larvae were treated by collagenase (Sigma no. C9891, 1 mg ml^−1^) in PBT for 10–30 min depending on the stage at room temperature. Larvae were washed three times for 5 min in H_2_O and then treated with acetone for 20 min at −20 °C. Animals were washed for 3 × 5 min in PBT. Animals were fixed again in 4% paraformaldehyde for 20 min and washed three times in PBT. Larvae were then incubated for 1–2 h in blocking solution (500 μl of 10% goat serum, 1% dimethylsulphoxide (DMSO), 1 × PBT) at room temperature under gentle agitation. Four hundred microlitres of the blocking solution were removed and one hundred microlitres containing primary antibody were added (Anti-Acetylated-Tubulin (Sigma-Aldrich no. T7451) and presynaptic anti-SV2 (Developmental Studies Hybridoma Bank) at 1:250 final dilution, anti-GFP (AMS Biotechnology no. TP401) at 1:1,000), and incubated overnight at 4 °C under slight agitation. Animals were rinsed three in PBT and washed for 4 × 30 min in PBT. Larvae were incubated for 1–2 h in 500 μl of the blocking solution (500 μl of 10% goat serum, 1% DMSO, 1 × PBT) at room temperature. Four hundred microlitres were taken out, and hundred microlitres containing secondary antibodies (Life Technologies Alexa Fluor 488 no. A-11034 and Alexa Fluor 594 no. A-21201) were added and incubated overnight at 4 °C. Samples were washed five in PBT and analysed and/or scanned using confocal microscopy^45^.

### Generation of *hsa-SMN1* plasmids

Using human cDNA as template, the ORF and a small 3′-UTR part of *hsa-SMN1* were amplified with the forward primer (63-hsa-smn-atg) and reverse primer (65-hsa-smn-reverse; Supplementary Fig. 1). The 976-bp PCR product was digested with EcoRI and XhoI and ligated in EcoRI/XhoI-digested and gel-purified pCS2+ vector. The corresponding plasmid was named pCS2+hsa-SMN1. In order to generate a tol2kit-compatible pME vector containing *hsa-SMN1*, pCS2+hsa-SMN1 was digested by EcoRI/BglII and the hsa-SMN1 cassette was gel-purified. This cassette was cloned into an EcoRI/BamHI-opened and gel-purified pME-MCS (237, from the tol2kit) plasmid, and the final construct was named pME-hsa-SMN1. pME-hsa-SMN1 was then combined with p5E-UBI (containing the ubiquitin promoter, a gift from Dr Benjamin Hogan) and p3E-polyA (302) in a pDestTol2CG2 (395) destination vector carrying cmlc2:GFP. The plasmid was named UBI:hsa-SMN1 and was used to generate transgenic lines in the rescue experiments.

### RNA and *miR-430* mimics injections

All pME-RNAi constructs were linearized with BspHI and purified before reaction. Sense GFP-miR RNA transcription was performed using the mMESSAGE mMACHINE SP6 transcription kit (Ambion), following the manufacturer's protocol. RNAs were purified using the megaclear kit following the manufacturer's protocol, aliquoted and stored at −80 °C. Injections were performed at the one-cell stage into the yolk and at 150 pg for all samples. pCS2+hsa-SMN1 was linearized with NotI. Sense *hsa-SMN1* RNA transcription and purification were performed as above. *hsa-SMN1* RNA was injected into the yolk of one- to four-cell-staged embryos at a concentration between 200 and 400 pg depending on the experiment. After injection, the remaining RNA was loaded on a gel to monitor RNA degradation. miR-430 mimics were obtained from Ambion (product ID MC10393) and were injected between 100 and 150 pg depending on the experiment.

### Imaging

Before analysis, animals were embedded in 1% low melting agarose. Images presented in this study were acquired using a Zeiss LSM710 confocal microscope coupled with the ZEN software. Images were processed under the image J environment when required.

RAW fluorescence was recorded using a Nikon microscope and the NIS elements software package. Image intensity measurements were performed in image J and processed through Excel software.

### Motor neuron phenotype quantification

In order to quantify motor neuron abnormalities, all animals were anaesthetized in tricaine and observed sideway under a fluorescent microscope, unless stated otherwise. Only CaP-axon projection abnormalities were scored. One point was attributed to every motor axon that showed defects as abnormal branching, abnormal length or absence of projection. Scores were compared using *t*-test. To validate the *in vivo* observations, immunostaining (using anti-GFP, anti-SV2 or anti-tubulin antibodies) was performed on fixed larvae as described above. The same scoring system was used.

### Survival assays

Adult zebrafish were pooled at the same time and mated for 1 h in order to generate synchronized embryos. Embryos were collected in E3 medium. When appropriate, the embryos were injected with RNA or miR-430 mimics (one-cell to four-cell stage). For every condition, the 20 brightest embryos (red fluorescence) were sorted on day 1 and used for the survival survey. Embryos/larvae were then counted twice a day in order to record death ratios over a period of 20 days. From day 0 to day 5, animals were stored in Petri dishes, and then transferred into beakers containing a small amount of paramecia until day 20.

### Behavioural assay

Behavioural analysis was performed using the Zebrabox (Viewpoint) following the manufacturer's instructions (http://www.viewpoint.fr/en/p/equipment/zebrabox). Zebrafish larvae were distributed in 24-well plates filled with E3 medium (one larvae per well). Plates were incubated at 28 °C in the dark for 1 h before analysis. The assay consisted of recording of larval behaviour during 10 min under light. At the end of the experiment, each larva was checked in order to exclude from the data potential dead animals. Data were exported and processed using Excel.

### Statistical analysis

Data were analysed using *t*-test. A minimum of three replicates was performed for each experiment, except when stated otherwise.

## Additional information

**How to cite this article:** Giacomotto, J. *et al*. Effective heritable gene knockdown in zebrafish using synthetic microRNAs. *Nat. Commun.* 6:7378 doi: 10.1038/ncomms8378 (2015).

## Supplementary Material

Supplementary Figures Supplementary Figures 1-11

Supplementary Movie 1 Representative video sample of behavioral experiment comparing control versus F2 UBI:miRsmn1-1#5 5dpf larvae

Supplementary Movie 2 Slow motion video showing 3-month old hsa-miR218-2:GFP (control) and smn1- knockdown hsa-miR218-2:GFP; UBI:miRsmn1-1#5 fish
