# Supplementary material for: Effective heritable gene knockdown in zebrafish using synthetic microRNAs
Source: Nat Commun. 2015 Jun 8;6:7378. doi: 10.1038/ncomms8378 (PMC4468906; doi:10.1038/ncomms8378)
Supplement: Supplementary Figures — 1-11 [file ncomms8378-s1.pdf]

SUPPLEMENTARY INFORMATION

RNAi		
NAME	TARGET	SEQUENCE
mCherry_Top01_miR155	5' start 362	TGCTGTCACCTTGTAGATGAACTCGCGTTTTGGCCACTGACTGACGCGAGTTCCTACAAGGTGA
mCherry_Bottom01_miR155	5' start 362	CCTGTCACCTTGTAGGAACTCGCGTCAGTCAGTGGCCAAAACGCGAGTTCATCTACAAGGTGAC
mCherry_Top02_miR155	5' start 485	TGCTGTCTGCTTGATCTCGCCCTTCAGTTTTGGCCACTGACTGACTGAAGGGCGATCAAGCAGA
mCherry_Bottom02_miR155	5' start 485	CCTGTCTGCTTGATCGCCCTTCAGTCAGTCAGTGGCCAAAACTGAAGGGCGAGATCAAGCAGAC
mCherry_Top03_miR155	5' start 588	TGCTGTTGATGTTGACGTTGTAGGCGGTTTTGGCCACTGACTGACCGCCTACAGTCAACATCAA
mCherry_Bottom03_miR155	5' start 588	CCTGTTGATGTTGACTGTAGGCGGTCAGTCAGTGGCCAAAACCGCCTACAACGTCAACATCAAC
mCherry_Top04_miR155	5' start 770 (3'UTR)	tgctgTAACCATGTCGCGTTGTGCTTgttttggccactgactgacAAGCACAACGACATGGTTA
mCherry_Bottom04_miR155	5' start 770 (3'UTR)	cctgTAACCATGTCGTTGTGCTTgtcagtcagtggccaaaacAAGCACAACGCGACATGGTTAc
mCherry04_Top01_miR30	5' start 770 (3'UTR) -miR30-	ggctagcaAAGCACAACttGCGACATGGTTActggtgcacatgatggagTAACCATGTCGCGTTGTGCTTc
mCherry04_Bottom01_miR30	5' start 770 (3'UTR) -miR30-	ggctgAAGCACAACGCGACATGGTTActccatcatgtgcaccagTAACCATGTCGCAAGTTGTGCTTtgct
mCherry04_Top01_miR218	5' start 770 (3'UTR) -miR218-	tcctTAACCATGTCGCGTTGTGCTTGGTGGAACGATGGAAACGGAAAAGCACAACGACATGGTTA
mCherry04_Bottom01_miR218	5' start 770 (3'UTR) -miR218-	TAACCATGTCGTTGTGCTTTTCCGTTTCCATCGTTCCACCAAGCACAACGCGACATGGTTActtt
smn1_Top01	smn1-3'utr	TGCTGTCCCGTTCATATCCCACTCGTGTTTTGGCCACTGACTGACACGAGTGGTATGAACGGGA
smn1_Bottom01	smn1-3'utr	CCTGTCCCGTTCATACCACTCGTGTCAGTCAGTGGCCAAAACACGAGTGGGATATGAACGGGAC
smn1_Top02	smn1	TGCTGTGTACAGGTTGCCATCTTCAGGTTTTGGCCACTGACTGACCTGAAGATCAACCTGTACA
smn1_Bottom02	smn1	CCTGTGTACAGGTTGATCTTCAGGTCAGTCAGTGGCCAAAACCTGAAGATGGCAACCTGTACAC
smn1_Top03	smn1	TGCTGATTTCTTGGATGCAGCAGCCTGTTTTGGCCACTGACTGACAGGCTGCTATCCAAGAAAT
smn1_Bottom03	smn1	CCTGATTTCTTGGATAGCAGCCTGTCAGTCAGTGGCCAAAACAGGCTGCTGCATCCAAGAAATC
smn1_Top04	smn1-3'utr	tgctgGAACAATTATGTCTCCAGCAGgttttggccactgactgacCTGCTGGACATAATTGTTC
smn1_Bottom04	smn1-3'utr	cctgGAACAATTATGTCCAGCAGgtcagtcagtggccaaaacCTGCTGGAGACATAATTGTTCc

Miscellaneous		
NAME	GENERAL COMMENT	SEQUENCE
78-Forw-xholdelete	to delete xhol in pDONR223	TCGAacgatagcatcgcagag
79-Rev-xholdelete	to delete xhol in pDONR224	TCGActctgcgatgctatcgt
72-SV40-Forw	PCR -SV40 from pCS2p+ (+EcorV)	AATTgataTCGTATTACGTAGATCCAGACATG
73-SV40-Rev	PCR -SV40fromf pCS2p+ (+EcorV)	AATTgatatCGAATTAAAAAACCTCCCACACC
41-dsREDForward	dsRED amplification for pME-642	ttttatcgatcgccaccatg
42-dsREDreverse	dsRED amplification for pME-642	attgcgatcgcctacaggaa
66-ceruleanforward	cerulean amplification for pME-643	ttaatttaaaccATGGTGAGCAAGGG
67-ceruleanreverse	cerulean amplification for pME-643	ttaaTTTAAATTACTTGTACAGCTCGTCCAT
80-M13extended	SP6 introduction in pME clone	CGTTGTAAAACGACGGCCAG
81-pME-SP6intoduction	SP6 introduction in pME clone	aattccatgGTGGCaGAGCTCCAAGTAGCTTGTATTCTATAGTGTCACCTAAATatcgatgcctgcttttttgtacAAAGTTGG
52_Forw_miR30	dre-miR30 amplification (bamHI)	atcaggatcccatcgattcgaa
53_Rev_miR30	dre-miR30 amplification (+bglII and XhoI)	aattctcgagtgcggccagatctggcgagagttcatcatatgaccagt
36_miR218Forw-5-BamHI	Amplification hsa-miR218 5' (+BamHI)	aattggatccCTCCTGTCCTCTCTCTGACG
37_miR218Rev-middle-SpeI-XhoI	Amplification hsa-miR218 middle (+BsmBI, SpeI and XhoI)	aattCTCGAGttggACTAGTgatcCGTCTCaAGGAAAGCCCCGCAGC
38_miR218Forw-middle-SpeI	Amplification hsa-miR218 middle (+SpeI and BsmBII)	aattACTAGTgatcCGTCTCaGAAAGCACCGTGCTCTCC
39_miR218Rev-3-BglII-XhoI	Amplification hsa-miR218 3' (+BglII and XhoI)	aattCTCGAGtgcggccAGATCTCCCTCTGTATCTGCATCCTTTG
63-hsa-smn-atg	*hsa-SMN1* for pCS2+	aattgaattcATGGCGATGAGCAGCG
65-hsa-smn-reverse	*hsa-SMN1* for pCS2+	ctcgagCGCTTCACATTCCAGATCTGT

Supplementary figure 1: List of primers/oligonucleotides used in this study.

>mCherry01-3UTRtarget
ATGGTGAGCAAGGGCGAGGAGGACAACATGGCCATCATCAAGGAGTTCATGCGCTTC
AAGGTGCACATGGAGGGCTCCGTGAACGGCCACGAGTTCGAGATCGAGGGCGAGGGC
GAGGGCCGCCCCTACGAGGGCACCCAGACCGCCAAGCTGAAGGTGACCAAGGGCGGC
CCCCTGCCCTTCGCCTGGGACATCCTGTCCCCTCAGTTCATGTACGGCTCCAAGGCC
TACGTGAAGCACCCCGCCGACATCCCCGACTACTTGAAGCTGTCCTTCCCCGAGGGC
TTCAAGTGGGAGCGCGTGATGAACTTCGAGGACGGCGGCGTGGTGACCGTGACCCAG
GACTCCTCCCTGCAGGACGGCGAGTTCATCTACAAGGTGAAGCTGCGCGGCACCAAC
TTCCCCTCCGACGGCCCCGTAATGCAGAAGAAGACCATGGGCTGGGAGGCCTCCTCC
GAGCGGATGTACCCCGAGGACGGCGCCCTGAAGGGCGAGATCAAGCAGAGGCTGAAG
CTGAAGGACGGCGGCCACTACGACGCCGAGGTCAAGACCACCTACAAGGCCAAGAAG
CCCGTGCAGCTGCCCGGCGCCTACAACGTCAACATCAAGCTGGACATCACCTCCCAC
AACGAGGACTACACCATCGTGGAACAGTACGAGCGCGCCGAGGGCCGCCACTCCACC
GGCGGCATGGACGAGCTGTACAAGTAAGACCCAGCTTTCTTGTACAAAGTGGgcatc
gaAAGCACAACGCGACATGGTTAtcgaAcgaccCAACTTTATTATACATAGTTG

>dre-smn-1
CATCAGCAGCTGCGTTCATCAGAGTGGCGCACGTTGGACTTGGGAAATATTTAAAAT
TTTCATACATCCCCATGGCCAATGGTGCAGAAGATGTCGTATTTTGTCGTGGGACTG
GTCAAAGTGATGATTCTGACATTTGGGATGATACAGCTTTGATTAAAGCATACGATA
AAGCTGTTGCATCATTCAAGAATGCTTTGAAAGGTGAGGATGGGGCGACCCCACAAG
AAAACGACAACCCAGGGAAGAAGAGGAAAAACAACAAAAAGAATAAGAGCAGGAAGA
GATGCAACGCAGCACCGGATAAAGAGTGGCAGGTTGGAGACTCCTGTTATGCGTTCT
GGTCTGAAGATGGCAACCTGTACACTGCCACCATTACCTCAGTCGACCAGGAGAAGG
GCACCTGTGTGGTCTTTTACACAGATTATGGAAATGAGGAGGAGCAGAACCTCAGTG
ACCTTCTGACTGAGCCTCCAGACATGGATGAAGATGCTCTGAAGACAGCAAATGTCA
AAGAAACAGAGTCCTCCACAGAAGAGAGTGATCGCTCTTTCACCCCACAGAAGTCCG
GTCATGCAAAGCACAAATCTAAAAGCAATTTTCCTATGGACCCCCATCATGGTTTC
CCAGTTTCCCACCTGGACCGCCACCACCACCACCCCACTTTAAAAAGATGGATGGCA
GACGAGGAGAAGGTCCTGGTCCTTCCTTTCCTGGATGGCCTCCCATGATTCCACTCG
GTCCACCGATGATCCCACCACCGCCACCTATGAGTCCAGACTTTGGGGAGGACGACG
AGGCTTTGGGCAGTATGCTGATTTCCTGGTATATGAGCGGCTATCACACTGGATACT
ATATGGGTTTAAGACAGGGCCGTAAAGAGGCTGCTGCATCCAAGAAATCGCATCGGA
AATAAAAACCCAACGAGTGGGATATGAACGGGATGCTGCTGGAGACATAATTGTTCT
TTTTTGTATCGTTTGTAAATAAATTAAAAGTAATTGTTCTCTTTTCA

XX START
XX STOP
XX TARGET

Supplementary figure 2: Sequences and miRNA-targets of i) mCherry expresses in transgenic fish used in figure 2 and figure 3, and ii) *dre-smn1*.

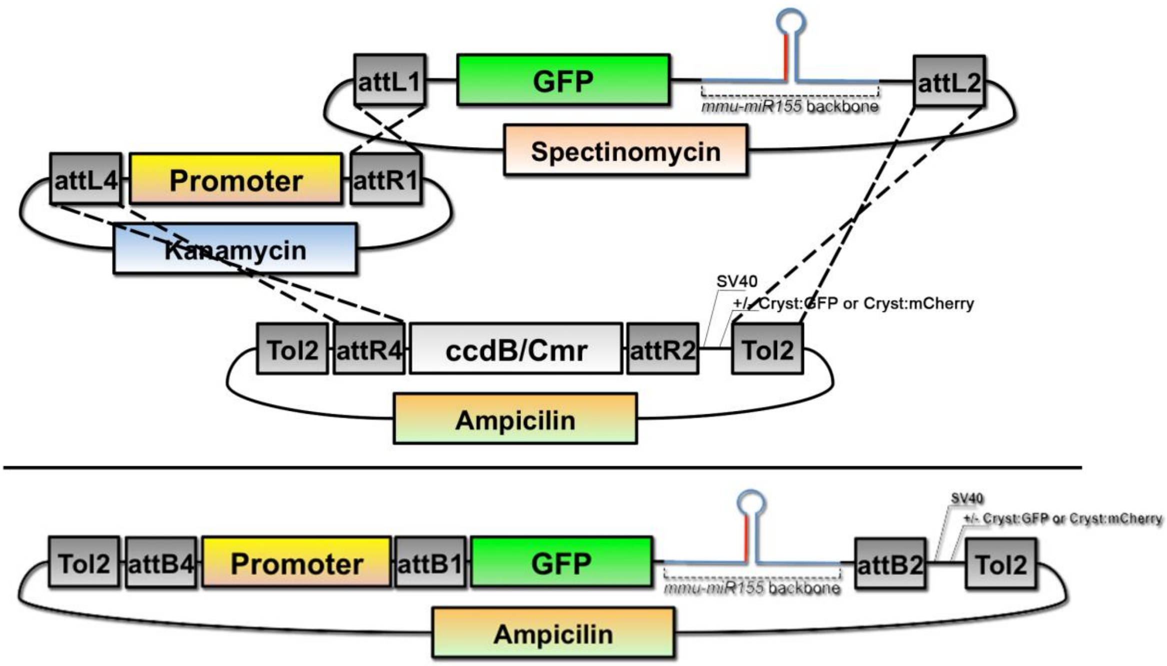

<u>Supplementary figure 3</u>: Schematic representation of multisite Gateway reaction used in this study to generate final miRNA-mediated knockdown tol2 constructs.

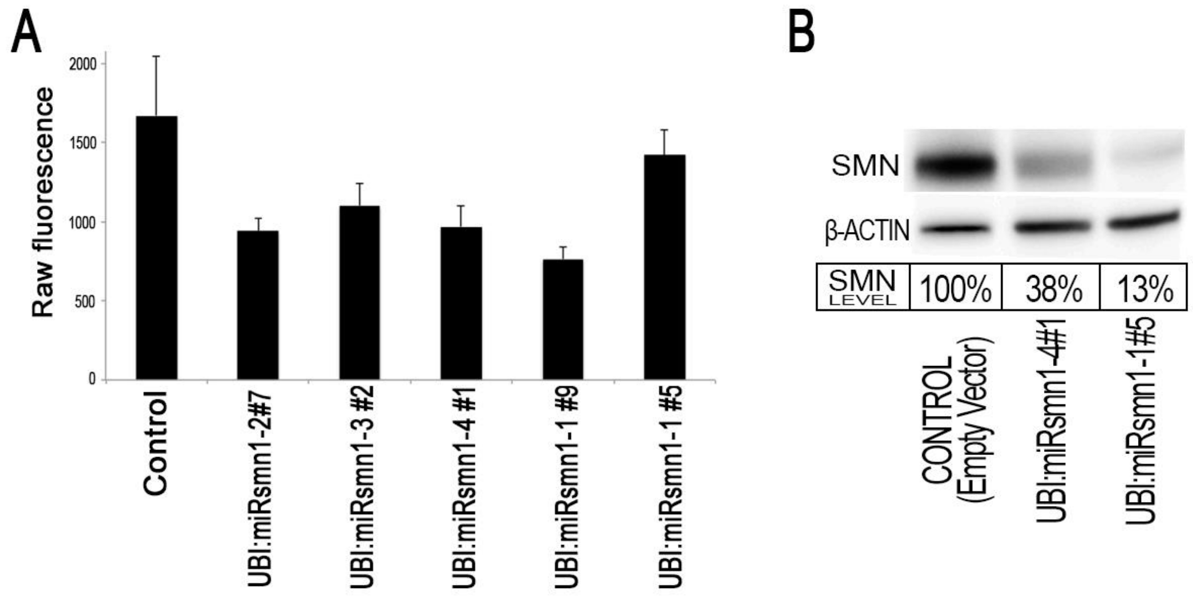

<u>Supplementary figure 4</u>: A, Raw red fluorescent (arbitrary units) of embryos presented in figure 6A. Means of 10 embryos ± standard deviation. **B,** Western blot of whole 6dpf transgenic larvae lysate revealing endogenous SMN protein in F2 UBI:miRsmn1-1#5 and F2 UBI:miRsmn1-4#1 versus control.

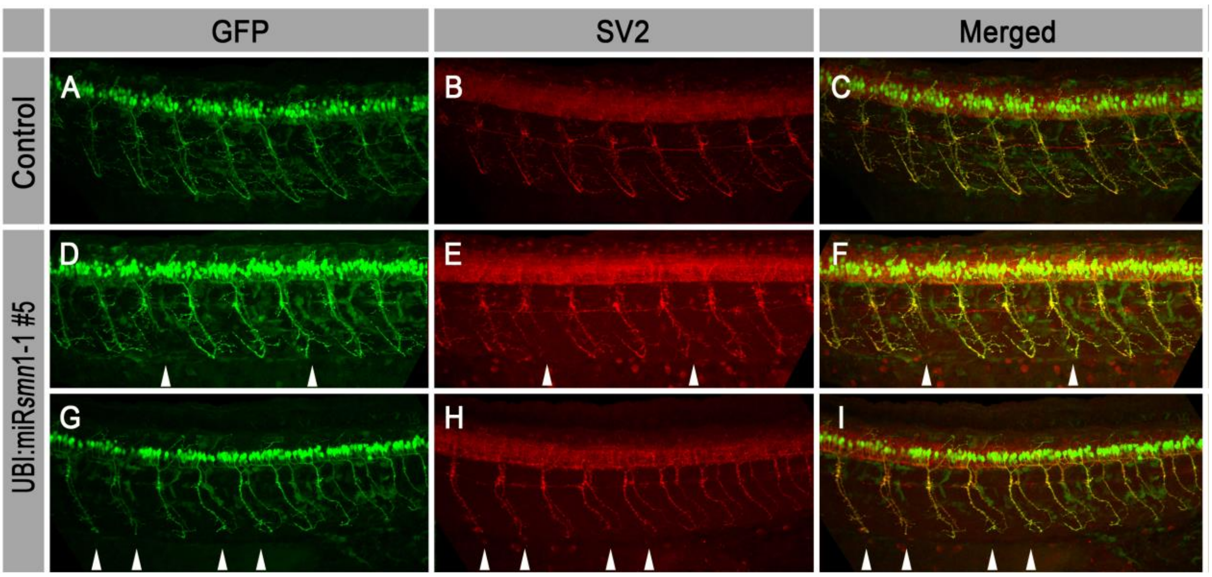

<u>Supplementary figure 5:</u> **Representative images of 52hpf hsa-miR218-2:GFP zebrafish larvae after immunostaining against GFP and SV2 proteins. A-C,** Lateral views of control (empty vector) larvae. **D-I,** UBI:miRsmn1-1#5 transgenic larvae expressing an artificial miRNA targeting the 3'UTR of *smn1* transcripts. Motor neuron abnormalities, such as short axons, abnormal branching and/or pathfinding errors, are labeled by white arrowheads.

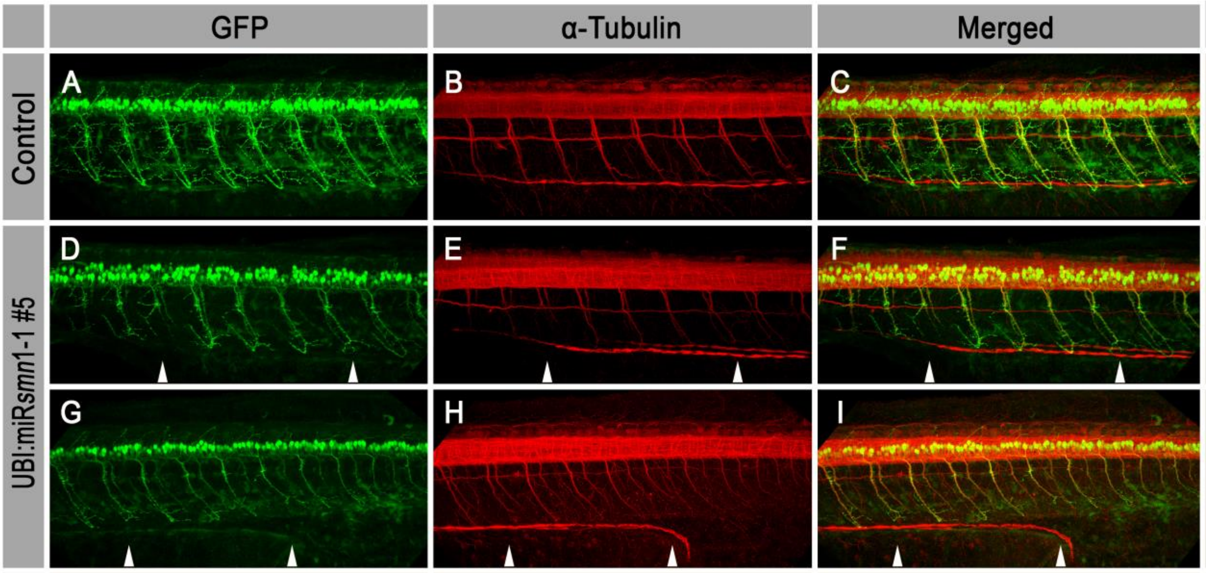

<u>Supplementary figure 6:</u> **Representative images of 52hpf hsa-miR218-2:GFP zebrafish larvae after immunostaining against GFP and α-tubulin proteins. A-C,** Lateral views of control (empty vector) larvae. **D-I,** UBI:miRsmn1-1#5 transgenic larvae expressing an artificial miRNA targeting the 3'UTR of *smn1* transcripts. Motor neuron abnormalities, such as short axons, abnormal branching and/or pathfinding errors, are indicated by white arrowheads.

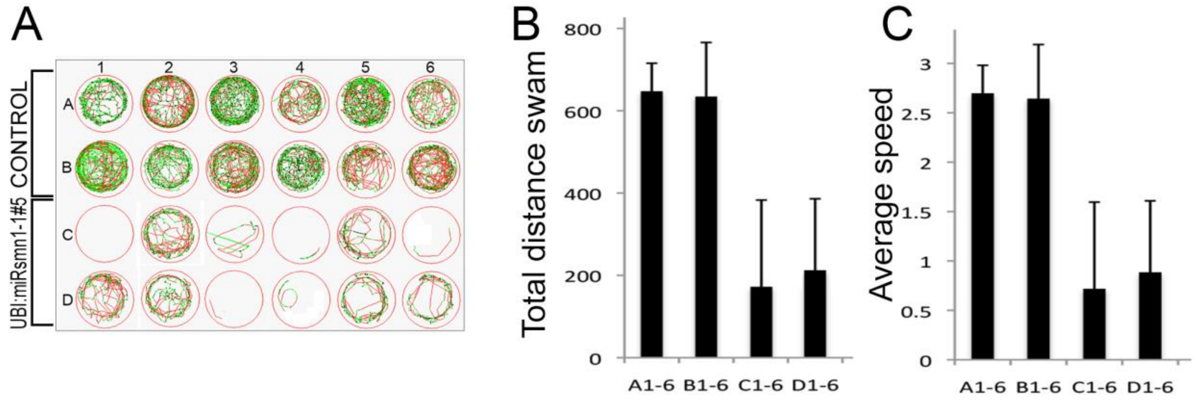

<u>**Supplementary figure 7:**</u> **Swimming behavior of control versus F2 UBI:miRsmn1-1#5 5dpf larvae.** Animals were loaded on a 24-well plate and swimming behavior was recorded for a period of 10 minutes under light. **A,** Schematic representation of the distance swum by each larva. **B,** Average distance (mm) swum by 6 larvae, ± standard deviation (one row). **C,** Average swimming speed (mm/sec) of 6 larvae, ± standard deviation (one row). See **videoS01** for a video sample related to this behavioral analysis.

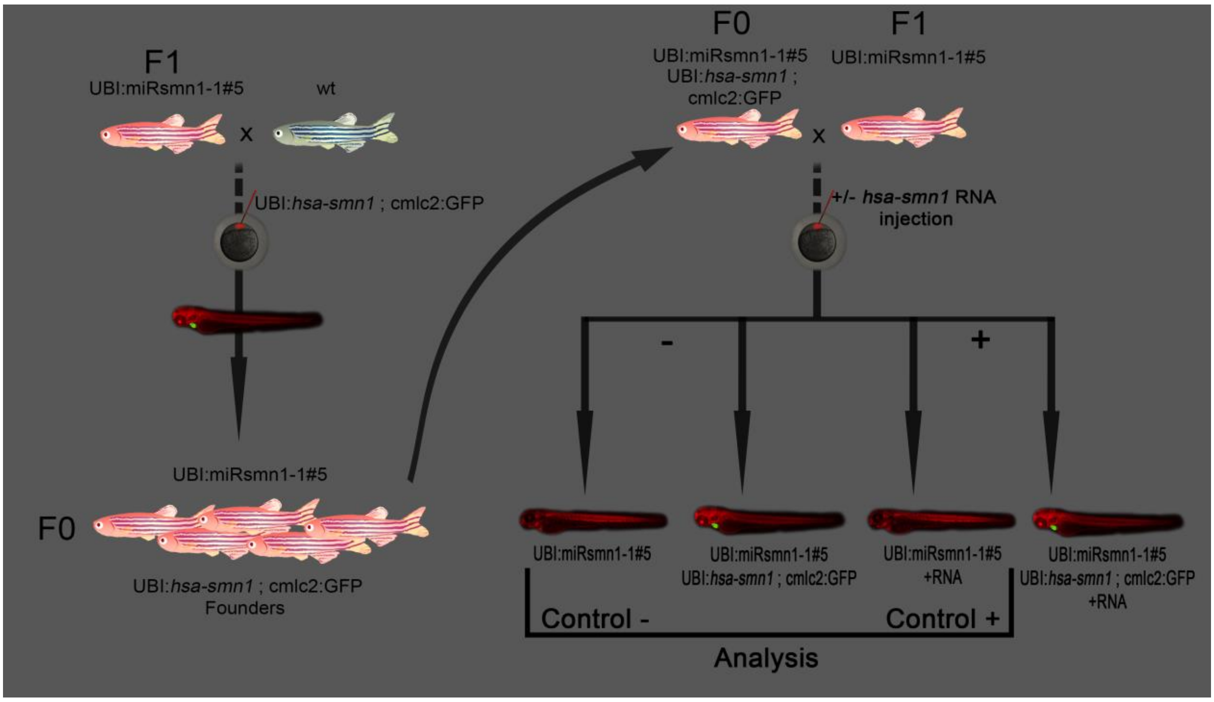

<u>Supplementary figure 8:</u> Schematic representation of the strategy followed i) to introduce *hsa-SMN1* into the UBI:miRsmn1-1#5 zebrafish line, and ii) to evaluate its capacity to rescue *smn1* miRNA-mediated knockdown phenotypes.

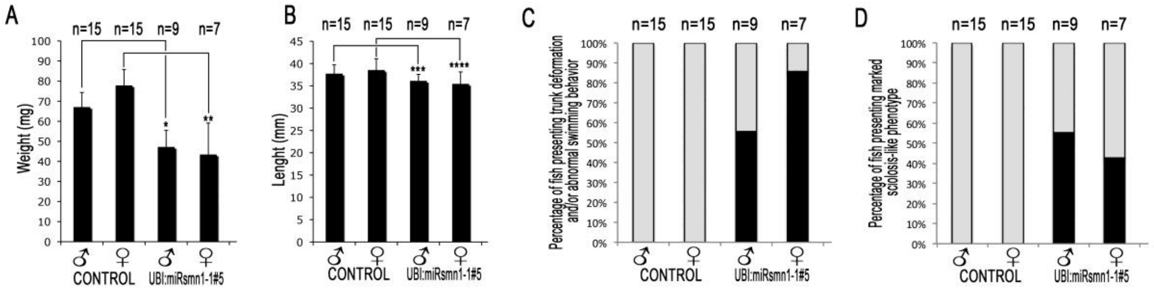

<u>**Supplementary figure 9:**</u> **F1 UBI:miRsmn1-1#5 animals develop late phenotypes, starting from 3 months post fertilization. A-C,** Summary of phenotypes recorded at 4 months post fertilization. In Comparison to control, F1 UBI:miRsmn1-1#5 fish present marked weight loss **(A)** and a slight overall size reduction **(B)**. F1 UBI:miRsmn1-1#5 fish also present with trunk deformities (such as scoliosis) and abnormal swimming behavior **(C & D and Video S02)**. Different from control at *0.001, **0.002, ***0.02, ****0.04.

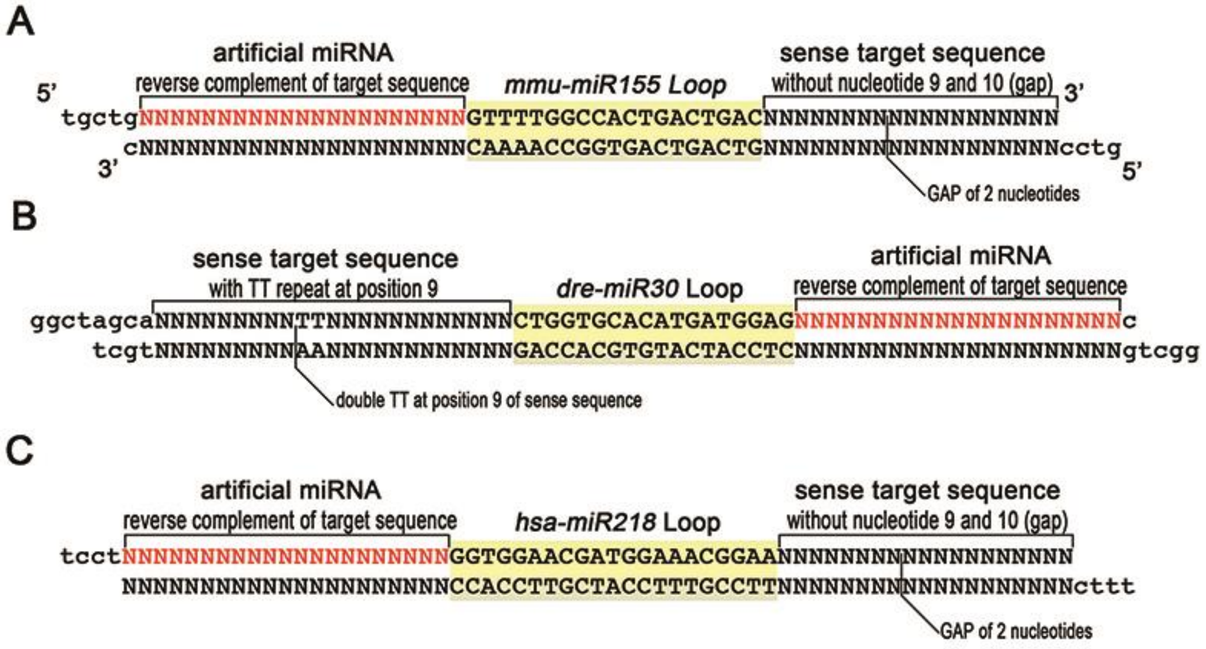

<u>Supplementary figure 10:</u> Artificial pri-miRNA design. A, Structure features of top and bottom oligonucleotides compatible with pME-RNAi651 plasmid digested by BsmBI. Sequence in red should be the perfect reverse complement of the gene's DNA target sequence. **B,** Structure features of top and bottom oligonucleotides compatible with pME-RNAi661 plasmid digested by BbsI. These oligonucleotides cannot be cloned directionally and sequence orientation has to be confirmed. **C,** Structure features of top and bottom oligonucleotides compatible with pME-RNAi661 plasmid digested by BsmBI. No additional modification or specific purification is required to anneal and ligate these oligonucleotides into their host plasmid.

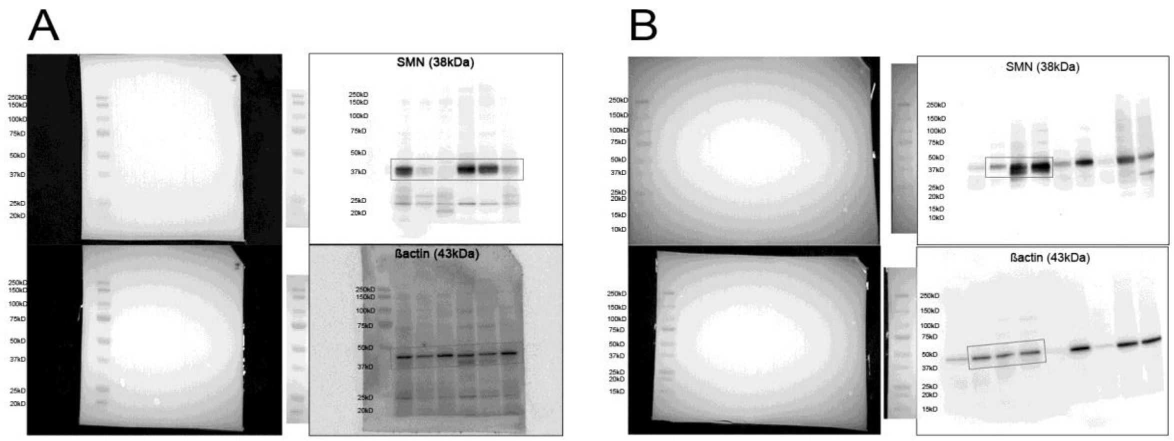

<u>Supplementary figure 11:</u> Uncropped images of Western blots presented in figure 4C and 7C. Molecular weight markers (kD) are indicated at left of each blot. Boxes indicate cropped images presented in figure 4C **(A)** and 7C **(B)**.
